# Socioeconomic inequalities in the utilization of dental services among adults in Saudi Arabia

**DOI:** 10.1186/s12903-022-02162-w

**Published:** 2022-04-21

**Authors:** Deema A. Sahab, Mohamed S. Bamashmous, Amitha Ranauta, Vanessa Muirhead

**Affiliations:** 1 Department of Dental Public Health, Faculty of Dentistry, King Abdulaziz University, Jeddah, Saudi Arabia; 2 Institute of Dentistry, Barts and The London School of Medicine and Dentistry, Queen Mary University of London, London, UK

**Keywords:** Dental services utilization, Socioeconomic inequalities, Adults

## Abstract

**Background:**

This study used the Anderson Behavioral Model to assess the socioeconomic inequalities in dental services utilization among adults in Saudi Arabia, along with other predictors of utilization, to inform future planning of dental care services.

**Methods:**

This cross-sectional study was a secondary analysis using national data from the 2019 Kingdom of Saudi Arabia World Health Survey (KSAWHS). The survey consisted of two interviewer-administered questionnaires: one household and one individual interview. The questions covered predisposing factors (age, gender, marital status, nationality, education, employment), enabling factors (income, household wealth, area-based socioeconomic class, health insurance, eligibility for free governmental health care, transportation and region of residence) and self-reported need for dental treatment. The main outcome was dental utilization in the past year; predisposing, enabling and need factors were independent variables. Hierarchical logistic regression analyses identified significant predictors of dental utilization, applying survey weights to adjust for the complex survey design. Adjusted odds ratios with 95% confidence intervals and *p* values were reported in the final model.

**Results:**

The final dataset included 8535 adults (response rate = 95.4%). Twenty percent of adults had visited the dentist at least once in the past year (95% CI 18–21%). There were socioeconomic inequalities in dental utilization. High household income (OR 1.43, *p* = 0.043), second and middle household wealth status (OR 1.51, *p* = 0.003 and OR 1.57, *p* = 0.006) and access to free governmental health care (OR 2.05, *p* = 0.004) were significant predictors in the final regression model along with perceived need for dental treatment (OR 52.09, *p* < 0.001).

**Conclusion:**

Socioeconomic inequalities in the utilization of dental services exist in Saudi Arabia. The need for treatment was the strongest predictor suggesting predominantly symptomatic attendance. Increasing awareness about the importance of preventive dental visits rather than symptomatic attendance could be an important policy implication to improve oral health and optimize dental care expenditure. Further research should explore the drivers for adults to seek preventive care in the absence of any recognized dental problems.

## Background

Oral diseases are the most prevalent noncommunicable diseases around the world and have substantial impact on health, societies, and economies [1]. The World Dental Federation (FDI) Vision 2030 report advocates for improving oral health and diminishing oral health inequalities over the next decade [2]. The FDI identified access to dental care and universal dental coverage as key pillars and global health priorities [3]. Improved dental healthcare services promote better work output (in education and employment) and help to alleviate impoverishment. Early detection of oral diseases reduces the expenditure on dental treatment; both have a direct positive impact on the overall quality of life [4].

### Dental health in Saudi Arabia

Increasing access to dental healthcare services and promoting prevention is also a priority in Saudi Arabia reflected by the National Transformation Program (NTP), designed to fulfil the Saudi Arabian Vison of 2030, which aims to improve the country’s public sectors and diversify its economy [5]. Saudi Arabia is a high-income country with a young population; the majority of its population is under the age of 40 [6, 7]. The burden of life-style related risk factors is rising in Saudi Arabia, especially among the young population [8]. Oral diseases contribute to this burden and account for up to 0.8% of daily-adjusted life years (DALYs) [9]. Dental caries and periodontal diseases are the most common oral diseases [10]. The prevalence of caries in Saudi Arabia is very high estimated to be around 80% based on local surveys [10]. Yet, the utilization of dental services is still relatively low despite the high prevalence of oral diseases [11]. Previous studies have shown that dental utilization among adults in Saudi Arabia ranged from 11.7 to 45.8% largely based on local or non-representative oral health surveys [11]. Dental services in Saudi Arabia are provided by the public and private sectors where all Saudi citizens have the right to free dental care in primary, secondary and tertiary government facilities [12]. Non-Saudi residents (38% of the population [13]) are not eligible for free dental services but have access to dental care through mandatory health insurance provided through their employers [14]. Previous studies have identified several reasons for participants not visiting the dentist including long waiting times [12, 15–17], limited available procedures [12, 18], dental fear [16, 17, 19, 20], lack of perceived need; expressed as having no pain or having no need for dental treatment [16, 17], and the high cost of treatment [15, 21]. These studies have investigated the predictors of dental utilization in Saudi Arabia. However, almost all of the published studies were localized to limited regions and targeted specific populations. None of these studies explicitly investigated socioeconomic inequalities in dental services utilization. This is an important knowledge gap recognized by a recent review [11] that begs the question: are there still socioeconomic inequalities in dental service utilization in Saudi Arabia even when a high proportion of the adult population has access to free dental care?

### Using the Andersen healthcare utilization model as a theoretical framework

The Anderson model of health care has been used as a theoretical framework to explore dental utilization and socioeconomic inequalities [22, 23]. This model suggests that predisposing characteristics, enabling resources and need factors shape the utilization of dental services [24, 25]. Predisposing characteristics comprise demographic factors such as age and gender [26], and social factors (e.g., education, ethnicity, and health beliefs) [27]. Predisposing characteristics affect the likelihood of using dental services through the natural history of oral diseases, genetic factors, health beliefs and social or cultural influences [28]. Enabling resources facilitate the use of dental services; income or wealth determines a person’s ability to pay for services, while insurance and cost-sharing rules define the actual price of the service and the amount that a patient pays out of pocket [29]. Need factors can be perceived need or evaluated need. Perceived need is how an individual evaluates their own health status, while evaluated need is an objective measurement of an individual’s health status often assessed clinically by health professionals [29].

Previous research that has used the Andersen model to assess socioeconomic disparities in the utilization of dental services has shown that enabling factors are key predictors [30]. Significant socioeconomic predictors included income [31–37], education [32, 33, 35–42], wealth [32, 41], dental health insurance coverage [32, 35, 40, 43, 44], and social support [28, 34, 43]. One study in Saudi Arabia used the Andersen model to investigate the utilization of dental services but only among children [45].

Income and education have been the main socioeconomic indicators used in previous Saudi Arabian research exploring dental utilization. However, education and income do not capture the full picture of how socioeconomic status impacts use of dental services in Saudi Arabia. Hence, there is a need to use multiple additional measures of socioeconomic status that are relevant in the local context [46], such as employment status, household wealth and region of residence [47]. Given that Saudi Arabia has a mixed dental system (offering free public and paid private services), many Saudi citizens use both private dental services, through dental insurance or out of pocket payment, and free governmental services. This could exacerbate socioeconomic inequalities in dental services utilization, since adults with higher income who can afford to pay for private dental services also have access to free governmental services [44].

Assessing the socioeconomic inequalities in utilization of dental services and the factors associated with them could help to inform strategies to reduce inequalities in access to dental care in line with the ongoing health system reforms. This study aimed to use the Andersen model to identify socioeconomic inequalities in dental services utilization and the predictors of dental services utilization by adults in Saudi Arabia.

## Methods

### Study design

In this cross-sectional study, we conducted secondary analysis using national data from the 2019 Kingdom of Saudi Arabia World Health Survey (KSAWHS) [48]. The 2019 KSAWHS was a national household survey led by the Ministry of Health to provide up-to-date estimates of priority health-related indicators. The survey gathered information from a nationally representative sample of 10,000 households covering several population health indicators including socio-demographic characteristics, use of dental services, self-reported oral health status, health insurance and access to health care services [48]. The data was collected between May and August 2019.

### Ethics

This study was a secondary analysis using data from the 2019 Kingdom of Saudi Arabia World Health Survey (KSAWHS). Ethical approval for the survey was obtained from the General Directorate for Research and Studies in the Saudi Arabian Ministry of Health. Participation was voluntary and informed consent was obtained from all participants. All methods were carried out in accordance with relevant guidelines and regulations.

### Sample selection and interview procedure

The survey followed a stratified three-stage sample design with a probability proportional to population size to obtain a representative sample of households and adults. The primary sampling units (PSUs) were census enumeration areas (EAs); geographic areas defined by the General Authority of Statistics (GASTAT) as part of the sampling process for collecting census data. In the second stage, a fixed number of eight households were systematically sampled from each PSU. Household heads completed the household interviews. The third stage then randomly selected a household member aged 15 years or older to complete the individual interview. For this study, only adults aged 18 years and above with complete outcome data were included in the analysis.

The survey consisted of two questionnaires (household and individual). All interviews were conducted at the respondents’ houses, by trained physicians or nurses. Data was collected by face-to-face interviews through Computer Assisted Personal Interview (CAPI) software using tablets.

### Measures

The survey included questions about socio-demographic characteristics, work history, household insurance coverage, household assets and income as well as health care utilization. These variables were mapped to the factors in the Andersen Model of Health Services Utilization [22]. Predisposing variables included age, gender, marital status, nationality, education, and employment. Nationality was dichotomized into Saudi and Non-Saudi. Respondents were asked about their highest level of education and their current employment status. Enabling variables were household income, region of residence (urban/rural), transportation, access to free governmental health care, health insurance coverage, household wealth index and an area-based measure of socioeconomic class. The area-based socioeconomic class indicator was adopted from a previous study that used Latent Class Analysis (LCA) to develop a categorical socioeconomic index using national census data and several household indicators mapped to an area: educational status, employment status, type of housing, tenure of housing, car ownership and material ownership [47]. The index classifies enumeration areas or governorates into four socioeconomic classes (1 = affluent class, 2 = upper middle class, 3 = lower middle class and 4 = deprived class) [47]. Index scores calculated from the study by Alomar et al. were assigned to the survey respondents in the KSAWHS sample. The household income was the monthly income of all household members in Saudi Riyals (SAR). Monthly household income was categorized into four groups following previous studies [49]: high household income (more than 15,000 SAR), upper-middle household income (10,000 to 15,000 SAR), lower-middle household income (5000–10,000 SAR) and low income (less than 5000 SAR). The place of residence was categorized as rural or urban based on the General Authority of Statistics (GASTAT) classification of their corresponding enumeration area. Transportation indicated if the household owned a car (Yes/No). Insurance coverage was determined if all household members were covered by mandatory, voluntary and/or free governmental health care. The wealth index is a composite measure developed by the Demographic and Health Surveys (DHS) program to evaluate a household’s overall living standard [50]. The scale developers used Principal Component Analysis (PCA) to generate a continuous scale of household wealth using a collection of household indicators such as house building materials, water and sanitation facilities and household ownership of assets (e.g., televisions and refrigerators). The scale was then divided into five wealth quintiles ranging from the 1^st^ quintile (lowest-poorest) to the 5^th^ quintile (highest-wealthiest) [50]. Perceived need was assessed asy self-reported oral health when the respondents were asked if they had any oral health problems in the past year.

Dental utilization was the primary outcome measure defined as the respondents seeing a dentist at least once in the previous year (Yes/No). This outcome included visits to either a government (free) dental service or a private dental clinic.

### Data analysis

Survey weights were calculated and applied to ensure representativeness of the data, taking into account the probability of selection at each sampling stage and adjusted for non-response rates at the three sampling stages [51]. The dataset had missing data on income, area-based socioeconomic class and self-reported oral problems, which is an inherent trait in most survey data [52]. Multiple imputation was used to replace missing values using chained equations and sensitivity analysis was conducted to determine the best approach (e.g., listwise deletion or complete case analysis). The sensitivity analysis showed similar results regardless of the method of imputation. All analyses were performed using the Stata survey design software package accounting for the complex sampling design and weighting. A hierarchical logistic regression was used to identify significant predictors of dental utilization outcomes based on the Andersen model. This involved first analysing the variables in a horizontal level separately by grouping the predisposing factors and enabling factors. The significant factors from each horizontal level analysis were identified using a 0.05 significance level. The significant variables from each level were then added to a final logistic regression model to adjust for all predictors. Adjusted odds ratios with 95% confidence intervals and p values were reported for the variables in the final model. The software used for all statistical analyses was Stata/SE for Mac (version 15.1, StataCorp LLC, College Station, TX, USA).

## Results

### Description of the sample

**Fig. 1:**
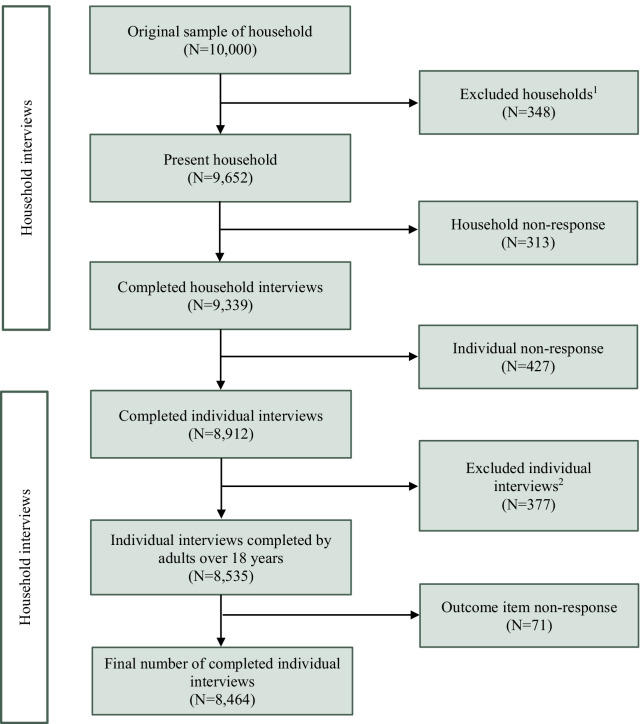
Number of completed household and individual interviews included in the analysis. ^1^Excluded based on the following interview result categories: household absent for extended period of time; Dwelling vacant; Address not a dwelling; Dwelling destroyed; Dwelling under construction; Dwelling status unknown. ^2^Excluded for the following reasons: 377 completed interviews were excluded because they were completed by individuals under the age of 18 years

Out of the 10,000 sampled households, 9652 were occupied, and 9339 completed the household interviews (household response rate = 96.8%). A total of 8912 respondents then completed the individual interviews (individual response rate of 95.4%). The final dataset excluded respondents who had missing outcome data and data from participants aged under 18 years leading to a final sample of 8464 respondents (Fig. 1).

Table 1 shows the demographic characteristics for the sample. Weighted data showed that 47% of the sample were female and 79% were under the age of 45 years, which mimics the Saudi population distribution [7]. Most participants were Saudi nationals (87%) who were currently married (70%), reflecting cultural and societal norms. The unemployment rate in the sample was 47%. This is higher than the unemployment rate in the population, which is 12% [53]. The percentage of unemployed respondents was higher for females (76%) than for males (22%) reflecting the family and home structure in the Arabic context where the most common reason for unemployment was being a homemaker or caring for family. Most participants resided in urban areas (86%), and almost half lived in affluent areas (48%). Eighty-eight percent of the respondents were eligible for free governmental health care and 23% were covered by health insurance. Only twenty percent of the respondents had visited the dentist at least once in the past year. Sixteen percent reported having problems with their oral health.

**Table 1:** Sociodemographic characteristics of survey respondents (*n* = 8464)

	Number of respondents	Percentage (unweighted)	Percentage (weighted)
*Gender*			
Male	4526	53.5	52.8
Female	3938	46.5	47.2
*Age*			
18–24 years	1278	15.1	16.6
25–34 years	3267	38.6	38.6
35–44 years	2097	24.8	23.6
45–54 years	952	11.3	11.1
55–64 years	505	6.0	6.1
65 + years	365	4.3	3.9
*Marital status*			
Never married	1658	19.6	21.9
Currently married	6200	73.3	70.1
Divorced/separated	290	3.4	4.0
Widowed	316	3.7	4.0
*Nationality*			
Saudi	7428	87.8	87.1
Non-Saudi	1036	12.2	12.9
*Education completed*			
No formal education	574	6.8	5.7
Less than secondary	1143	13.5	12.2
Secondary	2933	34.7	34.2
Diploma or formation	499	5.9	5.3
University	3054	36.1	39.1
Postgraduate	261	3.1	3.6
*Employment status*			
Not employed	3986	47.1	47.1
Employed	4478	52.9	52.9
Household income*			
Low income	1941	23.5	23.3
Lower-middle income	2811	34.0	32.1
Upper-middle income	1894	22.9	22.8
High income	1626	19.7	21.7
*Household wealth index*^†^			
Lowest quintile	2080	24.6	24.6
Second quintile	1815	21.4	21.8
Middle quintile	1622	19.2	19.1
Fourth quintile	1636	19.3	17.9
Highest quintile	1311	15.5	16.6
Area-based socioeconomic status*			
Deprived	115	1.4	1.2
Lower middle class	1067	13.2	10.3
Upper middle class	3994	49.3	41.1
Affluent	2928	36.1	47.5
*Place of residence*			
Rural	1414	16.7	13.9
Urban	7050	83.3	86.1
*Transportation (car ownership)*			
Yes	7664	90.5	90.4
No	800	9.5	9.6
*Eligibility for free governmental health care*			
Yes	7564	89.4	88.0
No	900	10.6	12.0
*Health insurance coverage*			
Yes	1746	20.6	22.9
No	6,718	79.4	77.1

^*^Variable has missing data that was managed using multiple imputation

^†^Composite measure calculated using data about ownership of consumer material such television and cars, household characteristics such as building material, source of drinking water, toilet facilities and other characteristics relevant to wealth status

### Predictors of dental utilization: predisposing factors

Adults aged between 35 and 44 years (OR 1.42, *p* = 0.006) were more likely to have visited the dentist in the past year compared to adults who were younger than 25 years (Table 2). Non-Saudi residents were less likely to have had a dental visit compared to Saudi residents (OR 0.62, *p* < 0.001) (Table 2). Currently employed individuals were less likely to have visited the dentist in the past year compared to currently unemployed adults (OR 0.82, *p* = 0.042) (Table 2). Gender, marital status, and level of education were not significantly associated with dental utilization (Table 2).

**Table 2:** Horizontal logistic regression analysis for visiting the dentist in the past year

	Adjusted OR [95% CI]	*p* value
*Predisposing factors*		
Gender		
Male	1.00 (ref)	–
Female	1.13 [0.94, 1.35]	0.20
Age*		
18–24 years	1.00 (ref)	–
25–34 years	1.10 [0.88, 1.39]	0.40
35–44 years	1.42 [1.10, 1.83]	0.01
45–54 years	1.27 [0.93, 1.74]	0.13
55–64 years	1.39 [0.93, 2.07]	0.11
65 + years	1.41 [0.87, 2.29]	0.16
Marital status		
Never married	1.00 (ref)	–
Currently married	0.87 [0.71, 1.07]	0.18
Divorced/separated	1.31 [0.90, 1.90]	0.15
Widowed	0.96 [0.63, 1.46]	0.86
Nationality		
Saudi	1.00 (ref)	–
Non-Saudi	0.62 [0.47, 0.83]	0.00
Education completed*		
No formal education	1.00 (ref)	–
Less than secondary	1.18 [0.78, 1.80]	0.43
Secondary	1.18 [0.78, 1.79]	0.42
Diploma or formation	1.22 [0.75, 2.01]	0.42
University	1.53 [1.00, 2.34]	0.05
Postgraduate	1.64 [0.95, 2.85]	0.08
Employment status		
Not employed	1.00 (ref)	–
Employed	0.82 [0.68, 0.99]	0.04
*Enabling factors*		
Household income*		
Low income	1.00 (ref)	–
Lower-middle income	1.07 [0.87, 1.32]	0.53
Upper-middle income	1.11 [0.87, 1.43]	0.40
High income	1.53 [1.18, 1.97]	0.00
Household Wealth index^†^*		
Lowest quintile	1.00 (ref)	–
Second quintile	1.52 [1.22, 1.89]	0.00
Middle quintile	1.40 [1.10, 1.77]	0.01
Fourth quintile	1.25 [0.96, 1.64]	0.10
Highest quintile	1.63 [1.23, 2.16]	0.00
Area-based socioeconomic class indicator*		
Deprived	1.00 (ref)	–
Lower middle class	1.57 [0.29, 8.56]	0.60
Upper middle class	0.98 [0.18, 5.21]	0.98
Affluent	1.10 [0.21, 5.90]	0.91
Place of residence		
Urban	1.00 (ref)	–
Rural	1.25 [0.94, 1.67]	0.12
Transportation (car ownership)		
No	1.00 (ref)	–
Yes	1.16 [0.85, 1.58]	0.35
Eligibility for free governmental health care		
No	1.00 (ref)	–
Yes	1.78 [1.29, 2.46]	0.00
Health insurance coverage		
No	1.00 (ref)	–
Yes	1.50 [1.24, 1.82]	0.00
*Need factors*		
Perceived need for dental treatment		
No	1.00 (ref)	–
Yes	52.03 [40.83, 66.29]	0.00

OR, odds ratio; CI, confidence intervals

^**†**^Composite measure calculated using data about ownership of consumer material such television and cars, household characteristics such as building material, source of drinking water, toilet facilities and other characteristics relevant to wealth status

^*^Data presented using test for linear trends

### Predictors of dental utilization: enabling factors

Adults from households with a high monthly income were over 50% more likely to have visited the dentist in the past year compared to those from households with a low monthly income (OR 1.53, *p* < 0.001) (Table 2). Adults from households with the highest wealth status had the highest odds ratio of seeing a dentist in the past year compared to adults living in households in the lowest wealth quintile (OR 1.63, *p* < 0.001) (Table 2). Adults who had access to free governmental health care (OR 1.78, *p* < 0.001) or who had health insurance (OR 1.50, *p* < 0.001) were more likely to visit the dentist during the last year compared to those who did not have access to free governmental care or health insurance (Table 2). There was no association between area-based socioeconomic status and dental utilization (Table 2).

### Predictors of dental utilization: need factors

Perceived need for dental treatment was a significant predictor in the horizontal level analysis (OR 52.03, *p* < 0.00) (Table 2).

### Predictor of dental utilization: final model

Eight variables were identified from the horizontal regression analyses as the most significant predictors of dental utilization and were included in the final (vertical) regression model. These variables were age, nationality, employment status, household wealth, household income, eligibility for free governmental health care, health insurance coverage and perceived need for dental treatment.

Table 3 shows the final regression model where perceived need for dental treatment remained the most significant predictor of dental utilization in the past year among adults in Saudi Arabia adjusting for predisposing and enabling factors (OR 52.5, *p* < 0.001). The odds ratio for household wealth was attenuated in the final model but remained a significant predictor. Adults living in households categorised in the second and middle household wealth index quintiles were more likely to have had a dental visit compared to adults from the lowest wealth quintiles (OR 1.51, *p* = 0.003 and OR 1.57 *p* = 0.006; respectively) (Table 3). Adults living in high income households had a higher probability of visiting the dentist compared to those living in households with low income (OR 1.43, *p* = 0.04) (Table 3). Adults who were eligible for free governmental health care (OR 2.02, *p* = 0.004) were two times more likely to have visited the dentist in the in the past year compared to those with no eligibility for free health care. Age, nationality (Saudi/non-Saudi) and insurance were no longer significant predictors in the final model adjusting for need and enabling factors (Table 3).

**Table 3:** Final logistic regression analysis for visiting the dentist in the past year

	Adjusted OR [95% CI]	*p* value
*Predisposing factors*		
Age		
18–24 years	1.00 (ref)	–
25–34 years	1.07 [0.77, 1.49]	0.67
35–44 years	1.31 [0.92, 1.86]	0.14
45–54 years	0.95 [0.65, 1.41]	0.81
55–64 years	1.07 [0.61, 1.88]	0.80
65 + years	0.69 [0.36, 1.33]	0.27
Nationality		
Saudi	1.00 (ref)	–
Non-Saudi	1.06 [0.70, 1.60]	0.79
Employment status		
Not employed	1.00 (ref)	–
Employed	0.82 [0.67, 1.00]	0.05
*Enabling factors*		
Household income		
Low income	1.00 (ref)	–
Lower-middle income	1.10 [0.84, 1.46]	0.48
Upper-middle income	1.11 [0.80, 1.55]	0.53
High income	1.43 [1.01, 2.02]	0.04
Household Wealth index^†^		
Lowest quintile	1.00 (ref)	–
Second quintile	1.51 [1.16, 1.97]	0.00
Middle quintile	1.57 [1.14, 2.16]	0.01
Fourth quintile	1.34 [0.96, 1.87]	0.09
Highest quintile	1.36 [0.94, 1.96]	0.10
Eligibility for free governmental health care		
No	1.00 (ref)	–
Yes	2.02 [1.25, 3.27]	0.00
Health insurance coverage		
No	1.00 (ref)	–
Yes	1.24 [0.94, 1.64]	0.12
*Need factors*		
Perceived need for dental treatment		
No	1.00 (ref)	–
Yes	52.45 [41.26, 66.69]	0.00

OR, odds ratio, CI, confidence intervals

^†^Composite measure calculated using data about ownership of consumer material such television and cars, household characteristics such as building material, source of drinking water, toilet facilities and other characteristics relevant to wealth status

## Discussion

This is only the second study to explore the utilization of dental services among adults in Saudi Arabia using a national survey including both Saudi and non-Saudi residents [54]. It showed low dental utilization despite the availability of dental services provided by the governmental and private sectors. Only 20% of adults above the age of 18 years visited the dentist within the past year. This concurs with previous studies that have reported reasons for Saudi citizens not using free dental services related to accessibility issues such as long waiting times [12, 15–17], limited available procedures [12, 18] and perceptions about higher quality care delivered in private clinics [12, 21]. The most common barrier preventing adults from using private dental services is the high cost of services [15, 19, 21]. Non-Saudi participants are more affected by the high cost of private clinics.

The findings showed that household wealth, household monthly income, eligibility for free governmental services and perceived need for dental treatment were significant predictors of dental services utilization in Saudi Arabia. The study used household wealth as a socioeconomic indicator, in addition to income and education, which is relevant to the Saudi Arabian context. The household wealth index measures aspects of socioeconomic status that are not captured by income but are critical for health outcomes. Several studies have showed that different social groups with similar incomes had significantly different wealth indices [46, 55]. Furthermore, material asset based indicators such as the household wealth index are more relevant in developing countries such as Saudi Arabia [50, 56].

Adults living in households with middle categories of wealth index were more likely to visit the dentist in the past year compared to adults in the lowest wealth index category even after adjusting for need. This conflicts with previous studies from other countries that reported socioeconomic inequalities in dental utilization in favor of individuals from wealthier households [41, 57]. This could reflect the conceptual nature of the wealth measure. Socioeconomic indicators that are based on the ownership of material assets vary depending on the context in which they were developed thus making it difficult to compare their results across studies [56]. This is the first time that researchers have investigated the association between household wealth and dental services utilization in Saudi Arabia. This study supports the relevance of wealth as a significant predictor of dental services utilization in Saudi Arabia.

Adults coming from high-income households were more likely to have a dental visit in the past year compared to those coming from low-income households. This finding is consistent with national and international research [11, 18, 58, 59]. Most adults living in high-income households are Saudi citizens who have access to both private dental services, through out-of-pocket payment or dental insurance, and governmental dental services. Non-Saudi residents struggle with the costs of dental treatment and are not eligible for free public dental care creating greater inequality in access to dental services [44].

Adults who were eligible for free governmental health care were more likely to have visited the dentist than adults who did not have free health care. This agrees with findings from previous studies [31, 42, 44]. However, there were still inequalities in dental service utilization despite free dental services, which suggests that providing free dental services may not lead to higher utilization rates. This study suggests that additional barriers may exist that require further research to inform the ongoing health care reforms [60].

Perceived need assessed by self-reported oral problems was the most significant predictor of dental utilization after adjusting for predisposing and enabling factors and despite only 16% of respondents reporting that they had an oral health problem. This underscores the importance of measuring need for treatment when assessing socioeconomic inequities health care utilization and the saliency of perceived need for treatment on health service utilization. Individuals with a perceived need for dental treatment were more than 50 times more likely to visit the dentist in the past year. Perceived need was previously reported as a predictor of symptomatic use of dental services [28, 45]. This is in line with Zola’s triggers that surmised that the nature and quality of symptoms was one of the five triggers that prompt individuals to use health services [61]. Perceived needs evaluated by patients can often differ from normative needs, which are objectively measured by clinicians [29]. Studies have also shown that individuals without a perceived need were less likely to have regular dental care [28, 62]. This raises an important question and policy implication for dental service planners in Saudi Arabia about whether the policy agenda should be to increase public awareness of the importance of regular preventive dental visits and/or develop dental services that support symptomatic use of dental services and urgent care [2]. In addition, in the context of Saudi Arabia where dental care services are provided under universal health coverage [63], some would argue that the financial burden of dental care makes preventive dental care a long-term cost saving option at the individual and provider levels [64, 65]. Others would support the need to promote both preventive visits and the use of urgent dental care.

Our study had several strengths. It was the second study to explore dental utilization using a national representative oral health survey sample providing a vital update from the previous national survey carried out in 2013 [54]. It was the first study to apply the Andersen Behavioral Model of Health Services Use to assess dental services utilization by adults in Saudi Arabia. It also contributed to the existing research by focusing on socioeconomic disparities in dental utilization among adults. The study had some limitations. It did not assess other known factors that may affect the utilization of dental services such as dental health beliefs and dental fear [66–68], and contextual factors such as community factors and amount and distribution of health care services [29, 32]. The study used a crude outcome measure to assess the utilization of dental services (Yes/No) with no regard for the reason of seeking dental care or information captured about utilization of private of government dental services. Previous studies found that the predictors of routine dental visits differ from predictors of visiting the dentist only when there is a dental complaint that needs treatment [21, 28, 54]. Hence, capturing information about symptomatic and preventive utilization could provide additional information about the demand for urgent dental care or preventive care among the adults in Saudi Arabia.

## Conclusion

This study confirmed the existence of socioeconomic disparities in the utilization of dental services among adults in Saudi Arabia despite free access to dental services. Adults with high monthly income, middle socioeconomic status and access to free governmental dental services were more likely to visit the dentist. However, perceived need for dental treatment was the strongest predictor of dental services utilization among adults. This suggests that adults’ utilization of dental services is mainly symptomatic, despite the availability of free dental care for the majority of adults in over 2000 primary health care facilities across the country [69]. Increasing the awareness of the importance of regular preventive dental visits as opposed to the symptomatic use of dental services is an important policy implication [2]. Our study highlighted the need for future research to explore the drivers for adults to seek preventive care in the absence of any recognized dental problems. This would facilitate the development of future policy initiatives aimed at enhancing the preventive use of dental services, diminishing oral health inequalities and optimizing dental care expenditure.

## Data Availability

The data that support the findings of this study are available from the Saudi Arabian Ministry of Health, but restrictions apply to the availability of these data, which were used under license for the current study, and so are not publicly available. Data are however available from the authors upon reasonable request and with permission of the Saudi Arabian Ministry of Health.

## Abbreviations

KSAWHS: Kingdom of Saudi Arabia World Health Survey
FDI: Fédération Dentaire Internationale (World Dental Federation)
PSU: Primary sampling unit
EA: Enumeration area
GASTAT: General Authority of Statistics
CAPI: Computer assisted personal interview
LCA: Latent class analysis
PCA: Principal component analysis
DHS: Demographic and Health Surveys
SAR: Saudi Arabian Riyal
